# Retinal Pigment Epithelium Cell Development: Extrapolating Basic Biology to Stem Cell Research

**DOI:** 10.3390/biomedicines11020310

**Published:** 2023-01-23

**Authors:** Santosh Gupta, Lyubomyr Lytvynchuk, Taras Ardan, Hana Studenovska, Georgina Faura, Lars Eide, Ljubo Znaor, Slaven Erceg, Knut Stieger, Jan Motlik, Kapil Bharti, Goran Petrovski

**Affiliations:** 1 Center for Eye Research and Innovative Diagnostics, Department of Ophthalmology, Institute for Clinical Medicine, Faculty of Medicine, University of Oslo, 0450 Oslo, Norway; 2 Department of Ophthalmology, Justus Liebig University Giessen, University Hospital Giessen and Marburg GmbH, 35392 Giessen, Germany; 3 Karl Landsteiner Institute for Retinal Research and Imaging, 1030 Vienna, Austria; 4 Institute of Animal Physiology and Genetics, Academy of Sciences of the Czech Republic, 27721 Libechov, Czech Republic; 5 Institute of Macromolecular Chemistry, Academy of Sciences of the Czech Republic, 16206 Prague, Czech Republic; 6 Department of Medical Biochemistry, Institute of Clinical Medicine, University of Oslo, 0372 Oslo, Norway; 7 Department of Ophthalmology, University of Split School of Medicine and University Hospital Centre, 21000 Split, Croatia; 8 Research Center “Principe Felipe”, Stem Cell Therapies in Neurodegenerative Diseases Laboratory, 46012 Valencia, Spain; 9 Institute of Experimental Medicine, Academy of Sciences of the Czech Republic, 11720 Prague, Czech Republic; 10 Ocular and Stem Cell Translational Research Section, NEI, NIH, Bethesda, MD 20892, USA; 11 Department of Ophthalmology, Oslo University Hospital, 0450 Oslo, Norway

**Keywords:** retinal pigment epithelium (RPE), differentiation, stem cells, cell therapy, age-related macular degeneration

## Abstract

The retinal pigment epithelium (RPE) forms an important cellular monolayer, which contributes to the normal physiology of the eye. Damage to the RPE leads to the development of degenerative diseases, such as age-related macular degeneration (AMD). Apart from acting as a physical barrier between the retina and choroidal blood vessels, the RPE is crucial in maintaining photoreceptor (PR) and visual functions. Current clinical intervention to treat early stages of AMD includes stem cell-derived RPE transplantation, which is still in its early stages of evolution. Therefore, it becomes essential to derive RPEs which are functional and exhibit features as observed in native human RPE cells. The conventional strategy is to use the knowledge obtained from developmental studies using various animal models and stem cell-based exploratory studies to understand RPE biogenies and developmental trajectory. This article emphasises such studies and aims to present a comprehensive understanding of the basic biology, including the genetics and molecular pathways of RPE development. It encompasses basic developmental biology and stem cell-based developmental studies to uncover RPE differentiation. Knowledge of the in utero developmental cues provides an inclusive methodology required for deriving RPEs using stem cells.

## 1. Introduction

**Figure 1:**
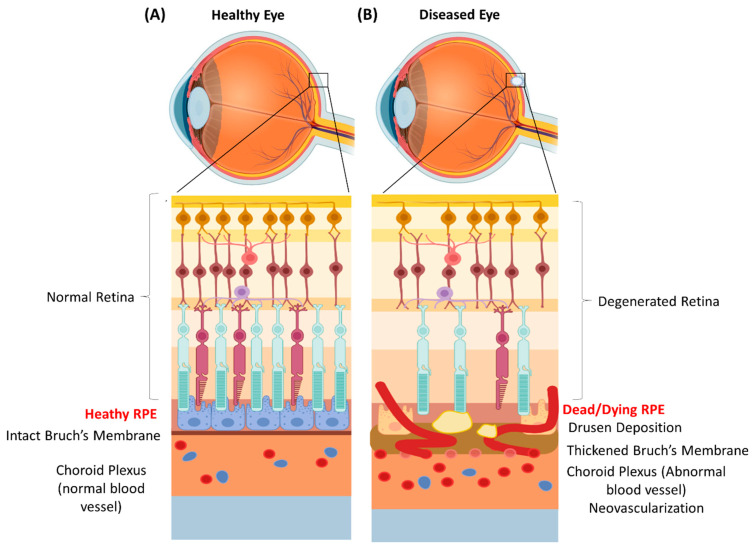
Schematic representation of the eye with structural and cellular organization of the retina with underlying retinal pigment epithelium (RPE). (**A**) Healthy eye with normal retinal cells and an intact RPE. The Bruch’s membrane is also intact with a normal underlying vasculature in the choroid plexus. (**B**) A representative eye with age-related macular degeneration showing degenerated and detached RPEs with the characteristic dead/dying RPEs. The Bruch’s membrane is damaged and increased neovascularization in the choroid plexus is observed. The figure has been created using BioRender (2023).

Age-related macular degeneration (AMD) is one of the most prevalent forms of irreversible vision impairment with a multi-factorial etiology comprising genetic, environmental, age-related, immune system-related, and lifestyle factors. Its pathogenesis is characterized by the early appearance of deposits of lipids and proteins (drusen), followed by functional anomalies in the retinal pigment epithelium (RPE) and progressive photoreceptor (PR) degeneration affecting the macular region [1] (Figure 1). These early stages are commonly referred to as the early or dry form of AMD. Choroidal neovascularization (NV) associated with fluid accumulation under the retina leads to the late or wet form of AMD. Geographic atrophy (GA) is another degenerative condition of the eye where the macula exhibits progressive degeneration starting from the perifoveal area and progressing to the fovea with time, eventually leading to permeant loss of vision. Late-stage AMD can be categorized by choroidal neovascularization of GA. GA is characterized by sharply defined area in the posterior pole with atrophy of the retinal pigment epithelium, the overlying photoreceptor, and the choriocapillaris [2].

Light-mediated ionization of photosensitive retinoids, generation of reactive oxygen species (ROS), and triggering of inflammatory cascade can initiate photoreceptor cell death [3]. Among the major factors leading to such death are aberrant choroidal neovascularization (CNV) [4,5], altered RPE phagocytosis [6], and subretinal recruitment of resident and monocytic microglia [7]. Functional as well as structural changes in the RPEs are thought to drive the onset of the disease [6,8]. The RPEs are a specialized pigmented cell monolayer, which is localized between the neural retina and the choriocapillaris. Degeneration and impairment of the RPEs results in the loss of vison as RPEs are associated with important functions, such as supplying nutrients to the PRs (rods and cones), phagocytosis, and maintenance of the retina –blood barrier, among others. Although the degenerative pathway of RPE cells and its association with AMD have been studied extensively, oxidative stress-induced cell death [9], complement pathway activation [10], dysfunctional mitochondria [11,12], and the underlying mechanisms of such dysregulations remain to be uncovered [6,13,14].

Currently, there is no therapeutic intervention that can fully reverse AMD [15,16]. However, treatment approaches, such as the intravitreal injection of anti-vascular endothelial growth factor antibodies (anti-VEGF agents), laser-based coagulation, and photodynamic therapy, are used to reduce the severity of wet AMD [17]. Apart from conventional therapeutic approaches, cell therapy-based regenerative approaches are currently being explored to treat AMD [18]. Stem cell-derived RPEs can be used for the derivation of allogenic or autologous RPEs (iRPEs) that can be injected in the subretinal space to replace the lost RPE cell monolayer and, thus, to attenuate/reverse the symptoms of AMD, including visual impairment [19,20,21,22]. The first study of ESC-derived RPE transplantation in human for testing its safety and efficacy in two patients with Stargardt’s macular dystrophy and dry age-related macular degeneration was published in 2012. The patients did not show any sign of hyperproliferation, tumorigenicity, ectopic tissue formation, or rejection after four months of follow-up. Thus, the results confirmed the short-term safety of transplantation of ESC-derived RPE in humans [23]. The first report of autologous iPSC-derived RPE in human studies was reported in 2017 where the authors transplanted iPSC-derived RPE sheets in a 77-year-old female patient with a subtype of neovascular AMD. A one-year follow-up showed that there was no improvement or reduction in visual acuity without any severe adverse reaction [24]. Currently, many clinical trials including iPSC are undergoing and the increase in iPSC-based RPE clinical trials over ESC underscores the utility of iPSC over ESC in deriving a long-term solution for cell therapy in AMD. There are few currently ongoing clinical trials which focus on patient-derived iPSC-RPE for treating dry AMD. Such clinical studies focus on autologous iRPE transplantation, thus advancing the iPSC-based cell therapy to an ideally personalized therapy.

However, recent studies suggested that the injection of RPE in the subretinal space did not lead to complete epithelization of the degenerated RPE monolayer and diminished reversibility of vision loss. This could be attributed to various reasons, including improper attachment of the RPEs on the underlying Bruch’s membrane (BM), cell clumping leading to the loss of cell-to-cell contact required for the formation of RPE monolayer [25,26], loss of photoreceptors, change in the functional behaviour of the transplanted RPEs in the various extracellular environments of the eye [27], and de-differentiation of transplanted RPEs leading to dysfunctional mature RPEs [15]. To overcome these limitations, transplanted RPEs seeded on a scaffold have been shown to improve the integration, regeneration, and functional behaviour in vivo when compared to scaffold-free cell transplantation. Scaffold fabricated using synthetic polymers have been explored in preclinical studies, such as PDLLA [28] and silk fibroin-polycaprolactone gelatin [29], and in clinical studies, such as parylene [30] and PLGA [31]. This knowledge further accentuates the need for newer scaffold-based delivery approaches, where a patch with pre-seeded RPEs could be directly transplanted in the subretinal space while preserving the functional property of the seeded RPEs and maintaining the function of the Bruch’s membrane 31]. This would overcome some of the limitations observed in the scaffold-free cell delivery approaches, including the de-differentiation of RPEs. Furthermore, this would help to properly engraft the transplanted RPEs with subsequent complete degradation of the scaffold. 

The first report describing in vitro development of RPEs involved spontaneous derivation of RPE-like cells in culture of ectoderm-committed embryonic stem cells (ESCs). This process usually takes 4–6 months to achieve sufficient number of cells for in vivo studies [15,19,20]. However, this process is spontaneous, and factors such as efficiency, gene expression, and functionality of RPE remain incompletely explored and may vary from batch to batch. In order to increase the efficiency of the differentiation strategy and improve the yield of RPE cells derived from ESCs and iPSCs, studies have been performed whereby the developmental process of the RPEs is recapitulated in utero and post-natally to develop differentiated RPEs in either a biphasic or a triphasic way [15,31,32,33]. The biphasic derivation strategy refers to the development of RPEs in two stages (anterior neuroectoderm and RPE specification and maturation), whereas the triphasic strategy refers to the derivation of RPEs in three stages (anterior neuroectoderm, RPE commitment specification, and maturation). However, the development of RPEs in utero is a more complex process and includes a multitude of signalling pathways and factors, both from the developing progenitor cells and the nearby extraocular mesodermal lineage cells, that contribute to the lineage-specific commitment and differentiation of the RPEs. Recapitulating such processes offers a potential strategy to derive functionally mature iPSC-based RPEs, which can be potentially used to develop a time-efficient protocol mimicking the developmental pathways of RPEs in utero. 

Therefore, in this article, we provide a concise summary of the developmental studies pertaining to the RPE using model-based developmental studies and stem cell-based cell signalling and developmental trajectory studies to uncover the mechanism of eye development through progressive stage-dependent commitment and progression. 

## 2. RPE Physiology and Function

The RPE is a tightly arranged monolayer of pigmented cells which has an important role in PR maintenance and visual function. The apical side of the RPE faces the PRs and regulates their function through their microvilli-like projections. These microvilli play an important role in the visual cycle between the RPE and the PRs by acting as a binding site for the photoreceptor outer segment (POS) and, thus, are essential for the phagocytic function of the RPE [6]. The basolateral side sits on a permeable membrane, i.e., the Bruch’s membrane, allowing the transports of nutrients from the choroidal blood vessels. The RPE has various roles including absorbing light that has been focused by the lens; transporting ions, water, and metabolic products from the retinal space to the capillaries of the choriocapillaris complex, and vice versa; and acting as an exchanger between the retina and the blood circulation [6,13]. The RPE acts as a recycling unit for phagocytosis of the PR outer segments (POS), returning metabolites to the PRs. The RPE additionally functions as an immune barrier and creates an immune privileged niche through its tightly regulated monolayer assembly and secretion of immunosuppressing soluble factors, commonly referred to as the blood–retina barrier (BRB). The RPE further helps in maintaining the structural functionality of the choriocapillaris network by secreting a variety of growth factors, such as VEGF. This helps in maintaining the physiological homeostasis by regulating capillary formation at the choriocapillaris plexus [16]. Therefore, irreversible damage to the RPE can lead to the development of various pathologies, including age-related macular degeneration, diabetic retinopathy, and Stargardt Disease [34]. It becomes imperative to understand the fundamentals of the developmental pathways of the RPE in utero, which can be essential for the development of therapeutic strategies to tackle RPE-related disorders. 

## 3. RPE Origin and Developmental Progression 

**Figure 2:**
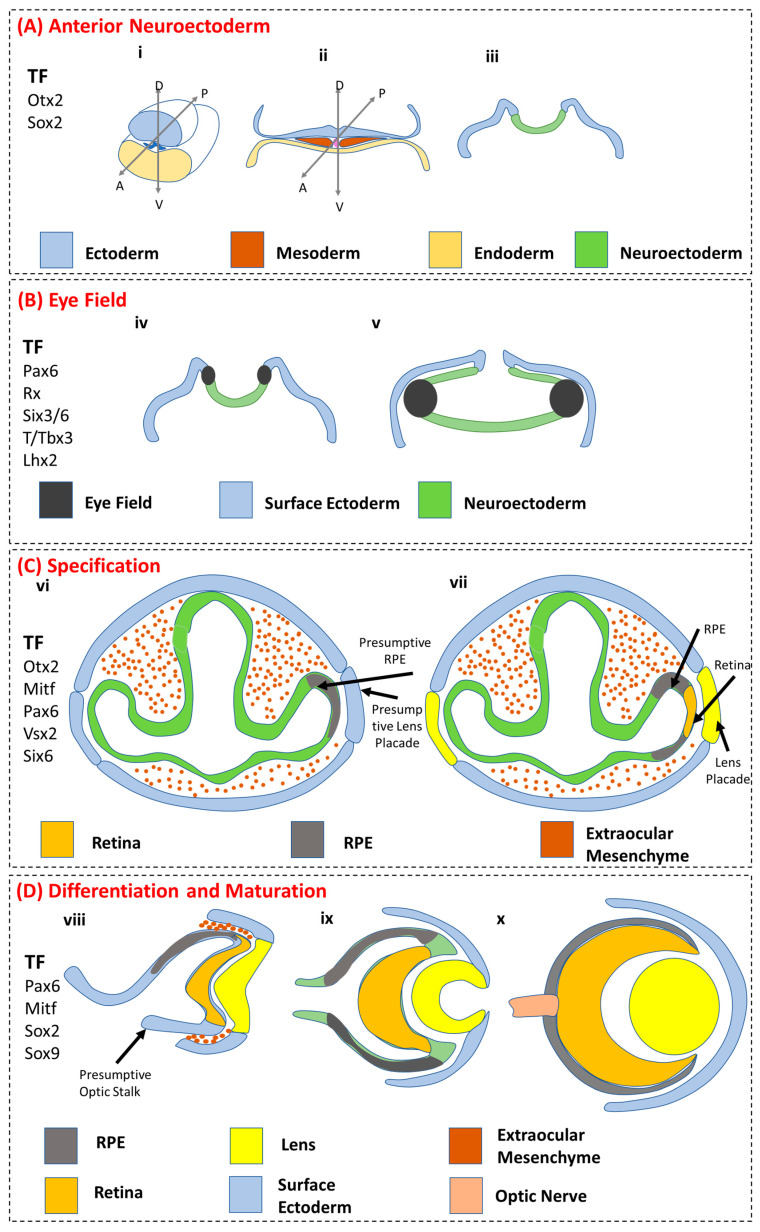
Schematic representation of the RPE origin and developmental progression. (**A**) Anterior neuroectoderm formation: (**Ai**) gastrulation leading to the formation of (**Aii**) three germ layers, i.e., ectoderm, mesoderm, and endoderm, and (**Aiii**) neurulation. (**B**) Eye field specification: (**Biv**) development of eye-field specialized cells, and (**Bv**) an expanding optic vesicle develops. (**C**) RPE specification: (**Cvi**) optic vesicle interaction with the surface ectoderm during the process of RPE specification, and (**Cvii**) with the further development of retinal specified cells. (**D**) RPE differentiation and maturation: (**Dviii**) concomitant development of RPE cells. (**Dix**) the outer layer becomes RPE. (**Dx**) The RPE monolayer and the retinal tissue layer form a single physical layer along with formation of the lens and the cornea.

The whole process of eye development is a complex mechanism that depends on multiple tissues of ectodermal and extraocular, mesodermal origins [35] (Figure 2). Here, we describe the stages of eye development and in particular RPE development as it occurs in utero. The whole process can be broadly categorized into four stages.

### 3.1. Anterior Neuroectoderm

Anterior neuroectoderm formation: Early embryonic development exhibits the formation of (Figure 2(Ai)) gastrulation, leading to the formation of (Figure 2(Aii)) three germ layers, i.e., ectoderm, mesoderm, and endoderm. RPEs arise from cells derived from the ectodermal and mesodermal layers, and (Figure 2(Aiii)) neurulation leads to the formation of the optic groove in the developing neural tube. The development of the eye involves a concentrated effort of three tissue sources, i.e., neuronal ectoderm, surface ectoderm, and extraocular mesenchyme. During the initial embryonic developmental stages of the eye, these tissue sources are derived from the neural crest and the mesoderm. Shortly after ectoderm formation, through the process of gastrulation, the eye field is specified in the medial anterior neural plate, containing all the cell progenitors of the neural crest-derived eye structures [36]. 

### 3.2. Eye Field

Eye field specification: (Figure 2(Biv)) The development of eye-field specialized cells arises from the anterior neural plate with an initial single eye field. This process further undergoes zonation to give two eye fields, leading to two eyes, as the optical groove region of the neural tube develops, and (Figure 2(Bv)) an expanding optic vesicle develops as the neural tube formation continues. The progenitor cells of the eye field express a set of transcription factors which are collectively known as eye-field transcription factors (EFTFs). These are PAX6, RAX, SIX3, and LHX2 [37]. The molecular effects of these EFTF expressions are not clearly understood due to their shared functionality in the forebrain development. However, studies have confirmed the critical role of these factors in eye field development. Fate mapping studies in *Xenopus* and zebrafish have demonstrated that progenitor cells in the eye field exhibit a complex yet coordinated migration and synchronous movement, resulting in the formation of the retina, the RPEs, and the lens [38,39]. The EFTFs exert many critical functions in regulating the development of the eye field and potentially also the development of the RPEs [37]. For example, the EFTF RX (in humans) regulates the changes in cell shape, proliferation, and segregation behaviour of progenitor cells in the eye field [40]. It also helps in maintaining eye field-specific gene expression. Studies manipulating the expression of RX have shown to produce eye formation anomalies, such as absence of the eye (anophthalmia), when RX is absent [41] or mutated [42]. Another important EFTF is SIX3 which has been shown to directly regulate the formation of RPEs. Induced loss of SIX3 results in defective patterning of the optic vesicle, which in turn results in the consequent development of the whole optic vessel into the RPEs [43]. Overexpression of SIX3 promotes ectopic optic vessel formation [44] and retinal development [45], while inactivation of SIX3 results in anophthalmia, along with other forebrain structures [44]. This underlines the essential function of these EFTFs in the early developmental stage of the eye field, which eventually leads to the development of the RPEs. 

### 3.3. RPE Specification

RPE specification: (Figure 2(Cvi)) The developing optic vesicle interacts with the surface ectoderm during the process of RPE specification. The RPE specified cells under the complex control of multiple cell-signalling factors develop on both sides (Figure 2 (Cvii)), with the further development of retinal specified cells and presumptive lens ectoderm during the course of eye development. Upon eye field development, evagination and formation of the optic vessel occurs in which intricate intracellular interaction controls the development of tissues and cells, including the retina, the RPE, the lens, and the optic stalk. The inner layer of the optic cup is directed towards the neural retina by receiving signalling from the overlaying surface ectoderm, whereas the outer layer develops into RPEs by interacting and receiving possibly activin-like signals from the extraocular mesenchyme [35,36,46]. This is the first time when functional diversification of RPEs occurs from the progenitors that arise from the eye fields during the process of eye development in the early developmental stage of the vertebrates [37]. It is at this stage when cells that are committed to the RPE lineage undergo two characteristic changes. The first change is the conversion from pseudo-stratified to monolayer cellular arrangement, and the second is the pigmentation of the cells, a characteristic phenotype that is exhibited by the RPEs throughout the life cycle of the organisms [46,47]. In mice, these features develop around embryonic day e11.5 and are the first explicit features of ocular differentiation that is maintained in the mature eye. The RPEs are essential for ocular development and morphogenesis of the eye and for proper retinal differentiation. 

### 3.4. RPE Differentiation and Maturation

RPE differentiation and maturation: (Figure 2 (Dviii)) The lens placode and the presumptive optical stalk develop with the concomitant development of RPE cells. The RPE specified cells undergo differentiation along with the development of the retina. The signalling factors from the extraocular mesenchyme play a role in the further development and differentiation of the RPEs: (Figure 2 (Dix)) the outer layer becomes the RPE and the inner layer becomes the retina along with the development of the lens pit (Figure 2 (Dx)). The RPE monolayer and the retinal tissue layer form a single physical layer along with the formation of the lens and the cornea, the latter developing from the surface ectoderm. In vivo differentiation of the RPEs appears to be influenced and regulated by a combination of key transcription factors (TF) which also have a critical role during the specification stage, including MITF, PAX6, PAX5, OTX2, SOX9, and LHX2. Notably, PAX6 is one of the TFs that plays a critical role in the RPE development during the early stages [48]. Its expression and regulation of other TFs (MITF and SOX9) further control the differentiation and maturation of the RPEs during the subsequent parts of the developmental process. It has been shown that PAX6 regulation of SOX9 controls the timely differentiation of RPEs along with choroidal vasculature development in mice [49]. PAX6 has also been shown to regulate the expression of a specific isoform of MITF, which is more critical for the RPE development than the other isoforms [50]. PAX6 and OTX2 play important roles in the pigmentation or melanogenesis of the RPEs [51]. Furthermore, PAX6 regulates the pigmentation of the RPEs through a forward-feed-forward regulatory interaction with MITF [52]. PAX6-mutant RPEs show defective pigmentation without affecting other RPE properties, such as the typical cuboidal morphology or the presence of tight junction among the RPEs 52]. PAX6 regulates the expression of MITF and activates the downstream gene expression of melanogenesis during the course of RPE differentiation post-RPE specification. Similarly, OTX2, besides controlling melanogenesis, also activates the retinol metabolism, an important pathway in the visual cycle, while SOX2 and OTX2 synergistically regulate the visual cycle genes [53]. The functionality of the visual cycle within the RPE confirms its maturation. Therefore, the expression and regulation of key TFs during the later stages of eye development form an essential mechanism regulating RPE differentiation and maturation. 

## 4. Genetic and Molecular Regulation of RPE Development and Differentiation

**Figure 3:**
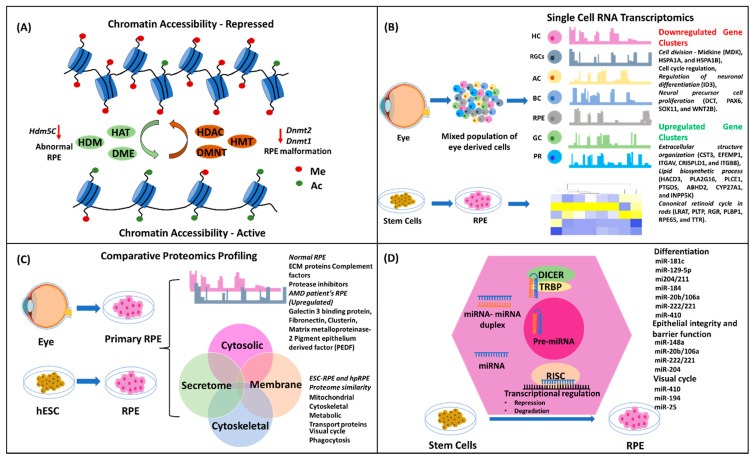
Schematic representation of the genetic and molecular regulation of RPE development and differentiation. (**A**) Epigenetic regulation controlling the RPE development. (**B**) Transcriptomics of the RPEs originating from primary cells isolated from humans, and stem cell-derived RPEs. (**C**) Comparative proteomic profile of the human RPEs and ESC-/iPSC-derived RPEs. Proteomes of various fractions (cytosolic, membrane, cytoskeletal, and secretome) display similarity when compared to primary and ESC-derived RPEs. (**D**) MicroRNA biogenies and their role in the development and differentiation of RPEs derived from stem cells. Various microRNAs have been shown to play a critical role in various stages of RPE development and physiological function. HDM—Histone demethylase; HDAC—Histone deacetylase; HAT—Histone acetylase; HC—Horizontal cells; AC—Amacrine cells; BC—Bipolar cells; PR—Rod and Cone PR cells; GC—Glial cells.

It is highly intriguing how such complex multicellular tissues and organs develop from a single cell. Biological explanation relates to the highly complex, spatiotemporally coordinated and synchronous activity of the multilevel regulatory systems, which include epigenetic, transcriptomic, proteomic, and microRNA regulation (Figure 3). In this section, we explain the critical role of each regulatory level and the important elements of each regulatory system in controlling the biogenesis, development, function, and maintenance of RPEs along with its implication in the development of AMD. 

### 4.1. Epigenetics 

An intricate, well-coordinated mechanism of early developmental cascade results in the development of the eye and related structures, including the RPEs. The epigenetic regulation, in turn, controls the genomic network in monitoring the lineage commitment, fate determination, and terminal differentiation of the RPEs. Consequently, any aberrant changes in the chromatin structure, either spontaneously or induced, manifest in developmental or age-related pathologies. Epigenetic regulation occurs at various levels of the chromatin assembly and, thus, offers a multitude of regulatory mechanisms responsible for highly coordinated lineage progression, fate determination, and differentiation. Such modulatory stages or levels include the chromatin remodeling of topologically-associated domains that are considered as the defined regulatory and structural units [54]. Another regulation is at the histone tail, which is modified either by the addition of acetyl and methyl groups via acetyl- and methyl-transferase, respectively, or by the removal of these groups by deacetylase and demethylase on the histone molecules [55]. Another well-known and highly important regulation at the DNA level results from the addition or deletion of the methyl group by a set of DNA methylating and demethylating enzymes [56]. In the case of RPE development, all these three levels of regulation play critical roles in the developmental pathways of the eye. Consequently, changes in the status of epigenetic markers are associated with RPE-related pathologies [57]. It is known that RPE and retina development is closely inter-dependent. A recent study in murine-derived RPEs showed that the epigenetic state of the RPE more closely resembles that of progenitor cell types, thus preventing the RPE from reprogramming and differentiating into retinal neurons. This claim was proposed based upon the evidence derived from a study about the repressive nature of chromatin structure in the promoter region of the non-PR retinal neuron gene and the highly methylated promoters of the PR genes [58]. 

The global profile of the chromatin accessibility in healthy RPE versus diseased RPE in AMD revealed a decrease in chromatin assembly in case of early AMD, suggesting RPE dysfunction is associated with AMD onset. In the same study, the correlation between a known risk factor (cigarette smoke) and AMD pathogenesis was shown to be similar in the RPEs derived from AMD patients and iPSC-derived RPEs. A global profile of chromatin accessibility using ATAC-Seq between these two groups showed that cigarette smoke-treated RPE recapitulated the chromatin accessibility changes as observed in AMD, thus providing an epigenetic linkage between a known risk factor for AMD and AMD pathogenesis. Overexpression of histone deacetylase (HDAC11) also resulted in decreased chromatin accessibility [59]. The important histone-modifying enzymes are histone demethylase and histone deacetylase. These enzymes remove the methyl and acetyl groups added to the lysine moiety of histones, respectively, and, therefore, regulate the repressive or closed state of the chromatin and control the gene regulatory networks for lineage-specific commitment and progression. A study in *Xenopus* showed that the knockdown of a lysine-specific histone demethylase 5C (Kdm5c) resulted in small and deformed systems with abnormal RPEs [60]. All these data suggest that epigenetic regulation not only plays a critical role in the early developmental stages of RPE, but also in RPE-related disease onset and progression.

Epigenetic conditioning or reprogramming has been used to reverse the age-related morphological and functional characteristics of the RPE. In a recent study, it was demonstrated that epigenetic regulation is highly critical not only during the course of RPE development in utero, but also throughout the life span of the organism [61]. The authors delivered OSK (Oct4, Sox2, and Klm) through an adeno-associated virus (AAV) in the subretinal space of aged mice. They observed that upon in vivo epigenetic reprogramming, the RPEs showed a reversal in morphology that was similar to that of young mice and exhibited reduced dysmorphic RPEs, reduced outer-segment accumulation, increased choriocapillaris fenestration, and reduced basal laminar deposits. Thus, it becomes highly essential to consider the epigenetic regulatory network and status of the genome, while designing strategies for RPE reverse engineering through the use of stem cells. In addition, studying the molecular basis of RPE development in utero and its association with other cell or tissue types arising from the same lineage during lineage commitment and fate determination at embryogenesis is also very important. 

The importance of the DNA methylating enzymes was shown when morpholino knockdown of DNA methyltransferase 2 (*dnmt2)* in zebrafish resulted in mild microphthalmia and RPE malformation [62]. In another study, conditional knockdown of *dnmt1* in mice resulted in abnormal RPE morphology and polarization at E15.5. Thus, this study underlines the importance of DNA modifying epigenetic regulatory enzymes in controlling the RPE apicobasal polarity and, in turn, regulating the development of the POS [63]. 

### 4.2. Transcriptomics 

Global transcriptomics of individual cell types enables the identification of a defined population of genes that play a critical role in the maintenance of the functionality and identity of individual cells. Previously, transcriptomics of tissue-derived cells was performed with inefficient isolation of pure cell type, leading to low-quality information. Single-cell-based analysis overcomes such challenges and presents a more realistic transcriptomic dataset of an individual cell type [64]. In a recent study, RPEs isolated from the human fetal eye were subjected to single-cell RNA-Seq analysis [65]. This study revealed that TFs, such as MITF, BHLHE40, OTX2, and SMAD3, were active in the RPEs compared to other cell types isolated from the retina, including glial cells, retinal ganglion cells (RGCs), and interneurons such as horizontal cells, amacrine cells, bipolar cells, and rod and cone PR cells. Upon clustering the variable gene, a total of nine gene clusters showed variable expression in the fetal RPEs. The downregulated genes included those involved in cell division, including Midkine (MDK), HSPA1A, and HSPA1B, cell cycle regulation, regulation of neuronal differentiation (ID3), and neural precursor cell proliferation (DCT, PAX6, SOX11, and WNT2B). The upregulated genes included those associated with extracellular structure organization (CST3, EFEMP1, ITGAV, CRISPLD1, and ITGB8), lipid biosynthetic process (HACD3, PLA2G16, PLCE1, PTGDS, ABHD2, CYP27A1, and INPP5K) and the canonical retinoid cycle in rods (LRAT, PLTP, RGR, PLBP1, RPE65, and TTR). The concomitant downregulation of the proliferative status of the cell and upregulation of the RPE-specific function-related genes demonstrated the transcriptomic profile of the in vivo developmental phase of human RPEs. Differential expression of visual function genes indicated that, during human eye development, and in particular RPE development, the visual cycle initiates by week 13 [65]. 

In another study using RPEs isolated from human donors, RNA transcriptomics using microarray revealed more genes associated with RPE function and physiology [66]. Functional annotation of the genes suggested that the RPEs exhibited high protein synthesis and high energy demand, and that the cells were exposed to a high level of oxidative stress and a variable degree of inflammation. Although the number of human donors were low (n = 6) in this study, the observation further underscores the existing knowledge that RPEs die in the age-related pathological manifestation of macular degeneration [66]. 

In another study using human pluripotent stem cells that differentiated into RPEs, transcriptomic profiling was performed at 1- and 12-month time intervals [67]. It was observed that with an increase in the culture time or the age of the RPEs, an increased expression of genes related to metal binding (MT1E, MT1F, MT1G, DCT, and MT2A) and anti-oxidant (DCT) function was observed. Human iPSC-derived RPEs showed similar maturation-associated transcriptional profile of RPE specific genes (PMEL, RLPB1, RGR, TYR, RBP1, and RPE65) and early retinal markers (PAX6 and RAX) compared to human primary RPEs (hpRPE) [68]. It was also shown that human iPSC-derived RPEs collected at different time intervals showed a common differentiation trajectory, indicating a more general mechanism of lineage specification and progression towards a functional RPE, albeit in a temporally differential manner. 

RPEs showed differential transcriptomic profile in the human eye isolated from the macula and from the retina periphery. Single-cell RNA sequence analysis of these two regions containing the RPEs from human eyes revealed that the macular RPEs had higher gene expression level of CXCL14, a gene that is associated with chemokine secretion and immune response regulation along with increased angiogenesis. The macular RPEs also showed higher gene expression levels of WFDC1, ID3, and CALCB, along with WFDC1. In the peripheral RPEs, increased expression of TFPI2, known to promote RPE proliferation, and IGFBP5, known to be associated with neovascularization, were observed. The peripheral RPEs also showed higher gene expression of GNGT1 and TFPI2 [68]. This study showed that the RPEs exhibited differential transcriptomic profile depending on the location within the retina and highlighted the importance of such information in designing therapeutic strategies. Another study with bulk retinal tissue showed a similar trend in the transcriptomic profile of the macula- and periphery-derived RPEs and choroidal tissue [69]. These findings emphasize the importance of cellular heterogeneity in transcriptomics and possible differential functional behaviour. 

### 4.3. Proteomics 

The first proteomic report of RPEs was published in 2003 by West et al. [70]. In their study, they isolated primary human RPEs from the normal eye and extracted proteins that were categorized into whole cell lysate, cytosolic fractions, membrane protein fractions, and microsomal fractions. A total of 278 proteins were identified comprising housekeeping proteins, proteins which are involved in the typical RPE function such as visual cycle, and enzymes associated with the retinoid metabolism. There have been refinements in studying proteomics at the single-cell level owing to advancements in the isolation and characterization techniques, such as nano HPLC-MS. The proteomics of the RPE can be categorized into two parts: the cellular fractions (intracellular, sub-organelle, and membrane proteins) which play an important role in the physiology and function of the RPE, and extracellular fractions (secretome and extracellular matrix (ECM) proteins) which maintains RPE paracrine-activity-based functions, such as maintaining choriocapillaris homeostasis and transporting vitamins across the BRB. Both the cellular and extracellular parts are essential and will be discussed further. 

Human ESC-RPE proteome has been compared to hpRPE proteome owing to the use of ESC-RPEs in clinical trials for the treatment of AMD [71]. Upon total protein comparison, 1041 common proteins were found to be similarly expressed and regulated in ESC-RPEs and hpRPEs. The ESC-RPE proteome displayed a similar profile when compared to the hpRPE proteome, covering a variety of compartmental subcellular fractions including mitochondrial, cytoskeletal, metabolic, and transport proteins. The critical proteins of the hpRPE proteome covered functions such as visual cycle and phagocytosis, and were found to be similarly expressed in the ESC-RPEs. 

Another investigation compared the proteome of human fetal primary RPEs (hfRPE) to that of an established RPE cell line, i.e., ARPE-19 [72]. The authors specifically compared the transmembrane proteome including proteins involved in drug and nutrient transport, such as ATP-binding cassette (ABC) and solute carrier (SLC) transporters. Out of the 41 target proteins, 16 proteins were expressed in the hfRPE and 13 in the ARPE-19 cells. There was a 4-fold difference in the expression of MRP1, MRP5, GLUT1, 4F2hc, TAUT, CAT1, LAT1, and MATE1 proteins between the hfRPE and the ARPE-19 cell line. HfRPEs showed a detectable level of MPR7, OAT2, and RFC1, whereas these proteins were below the detection level in the ARPE-19 cell line. The latter showed higher level of expression for MCT1, MCT4, MRP4, and Na^+^/K^+^ ATPase with a 4-fold difference compared to hfRPEs. Overall, this study showed that membrane proteins had variable levels of protein expression, but the pattern was similar between hfRPEs and ARPE-19 cells albeit not identical. This study also demonstrated that using established cell lines for transplantation purposes needs to be evaluated at the proteomic level, as the proteins eventually perform the catalytic activity and functional role in any given cell. However, it is to be noted that the ARPE-19 cell line may not be considered an ideal cell model to study the RPE due to its differential transcriptomics, absence of lower expression of several core RPE-related proteins, and display of non-differentiated phenotype, among other reasons [73]. 

Continuous long-term culturing of the RPE leads to a loss of functions and cell de-differentiation [74]. Such de-differentiation processes induce a change in the proteomic profile. In a recent study, the authors compared the differential protein expression profile of a hpRPE, a de-differentiated RPE, and a proliferative RPE [75]. They found that in the hpRPE, there were proteins with highly specialized functions related to the interaction with PRs, such as RPE65, CRALBP, and CRBP. Such proteins were absent in the de-differentiated RPE. The latter showed the expression of proteins associated with cell shape, cell adhesion, and stress fiber formation, including cytokeratin 19, gelsolin, and tropomyosin. Such change in the proteome of the native and de-differentiated RPEs reflects the physiological and functional state of the RPEs, and the information could be used for understanding and optimizing the culture conditions for RPEs for research and translational purposes. 

The extracellular proteome offers a very important and insightful role in the physiology, function, and interaction with neighbouring cells. It includes the secretome in the form of secreted proteins, exosome-containing proteins, and ECM proteins that form the microenvironment of the cellular niche and help maintain the morphological state of the cell. In another study, the global-scale proteome secreted from RPEs was studied to understand the molecular mechanism of AMD pathogenesis in the context of drusen formation in the eye [76]. The experiments included normal human primary RPEs and RPEs isolated from AMD patients and age-matched controls. They found that the normal RPEs showed secretion of ECM proteins, complement factors, and protease inhibitors [76]. These secreted proteins have also been associated with drusen formation, a hallmark deposit in AMD. Interestingly, the RPEs from AMD patients showed higher-fold expression of secreted proteins, such as galectin 3 binding protein, fibronectin, clusterin, matrix metalloproteinase-2, and pigment epithelium derived factor (PEDF), compared to the age-matched normal donor RPEs. Overall, this study generated important information about the secretome and its quantitative difference between age-matched normal RPEs and AMD patient-derived RPEs, as well as highlighted a mechanistic role of the secreted proteome in the formation of drusen deposits. 

### 4.4. MicroRNA

Micro RNA, commonly abbreviated as miRNA, was first identified in *C. elegans* as a small class of non-coding RNA molecules (typically 20–25 nucleotides) that regulate the expression of genes at the post-transcriptional level [77,78]. The mechanism of miRNA-based mRNA expression is well characterized by the binding of miRNA to a specific sequence segment in the mRNA, thereby controlling or regulating gene expression. For a detailed understanding of the biogenesis and mechanism of miRNA action and cellular pathway regulations, readers can follow the reviews by Ha et al. [79] and Trieber et al. [80]. The role of miRNAs has been shown to be very critical in RPE development and biogenesis. Studies examining the variability and dynamics of miRNA expression during the process of differentiation of ESC to functional RPE showed a stringent correlation between the types of miRNA expression and the stages of RPE differentiation [81,82]. The first study utilized the spontaneous differentiation ability of embryoid bodies of iPSC to form pigmented epithelial cells or the retina. The miRNA profile confirmed that miR-181c and miR-129-5p were upregulated and might have played a role in the differentiation process [81]. The essentiality of miRNAs and their biogenesis in RPE development were shown by conditional knockdown of miRNA biogenesis enzymes, such as Dicer1 [83], Drosha, or Dgcr8 [84]. In a study where Dicer1 conditional knockdown in mice was created under the control of the Cre-Lox delivery system [85], it was observed that the RPE cells’ morphology was smaller and depigmented, and that they failed to express critical enzymes involved in the visual cycle [86].

There is a large number of miRNAs that have been identified across species, and these play critical roles in the developmental pathways and functionality of RPEs both in utero and during post-natal development [87]. One group of miRNAs was found to have a horizontal role in species such as frog, fish, and mice is the 204/211 family of miRNAs, and it had been studied extensively. Studies in hfRPEs have shown that transfection with miR204/211 promotes differentiation into mature RPEs, while inhibition of miR204/211 shows de-differentiation of hpRPEs. The direct interaction of miR204/211 with MITF shows its positive role in the maintenance of the RPE phenotype [88]. Thus, miR204/211 could also be used as a potential therapeutic candidate in preventing de-differentiation of RPEs and promoting functionality. Other miRNA candidates that have been shown to directly regulate the differentiation of RPEs are miR-184 [89], miR-20b/106a, miR-222/221 families [86], and miR-410 [90]. 

Apart from playing a role in the differentiation process both in utero and in vitro, it has been shown that miRNAs regulate cell-to-cell interaction and membrane transporter proteins. Thus, miRNAs are involved in controlling one of the critical features of the RPE, which is the maintenance of its polarity and monolayer arrangement with highly packed and tightly regulated cell-to-cell connections. This feature also helps in maintaining the BRB and in regulating the transport of ions and molecules across the choriocapillaris and the retina [91]. MiR-204 has been shown to promote epithelial barrier formation in RPE [92]. Other miRNAs, such as miR-148a [93], miR-20b/106a, miR-222/221 [86], and miR-204 [94] have been shown to play a direct role in regulating and controlling the epithelial barriers and membrane transport in the RPE, thus maintaining its epithelial integrity and barrier function. 

The role of the RPE in the visual cycle is very important. Otherwise, the PRs would die rapidly due to the accumulation of photo-oxidized protein products as a process of light adsorption and transmission of signals. This cycle involves the development of POS which is phagocytosed by the RPE. Inside the RPE, all photo-oxidized proteins and phospholipids of the outer segments are processed, recycled, and transported back to the PRs. The process of phagocytosis and cell clearance have been shown to be regulated by a group of miRNAs, such as miR-184. It was observed that inhibition of miR-184 resulted in lowering the phagocytosis of POS and the recycling process. A dysfunction in phagocytosis or an imbalance in the visual cycle-related recycling of POS has also been implicated in the development of AMD. It was further confirmed that the RPEs isolated from AMD patients showed a downregulation of miR-184, thus pointing out the role of this miRNA in the visual cycle as well as AMD pathogenesis [95]. Similarly, several miRNAs have been implicated or associated with visual cycle recycling. They include miR-410 [96], miR-194, [97] and miR-25 [98]. Treatment of umbilical cord-mesenchymal stem cell-derived RPEs with miR-410 in vitro [96] and injection of miR-25 in the subretinal space of a sodium iodate-induced retinal degeneration rat model [98] improved the phagocytic capability of the RPEs and the degeneration of the RPEs in rats, respectively. 

## 5. Significance of the Developmental Studies

Developmental studies of the eye using animal models and pluripotent stem cells have helped in elucidating the pathways responsible for stage-specific progression of various eye tissues [99]. The findings that the RPE is required for retinal development and that retinal tissue is required for RPE development is essential [53]. This reveals the larger question of multi-signalling pathways and cellular dependence on RPE tissue development. However, the protocols that exist so far for RPE differentiation utilise few although critical signalling pathways that have been associated with stage-specific development of RPE in utero. For example, the first stage of RPE development is the derivation of anterior neurectoderm (ANE). Although variability exists, the majority of the protocols utilised dual SMAD inhibition (TGF and BPM) of iPSC/ESC to induce forebrain or ANE development [31,100,101,102]. However, the development of ANE does not rely solely on dual SMAD inhibition, although it is essential for ANE formation. A study by Greber et al. showed that ANE development can be efficiently induced by inhibiting the FGF signalling pathways along with dual SMAD inhibition [103]. It should be noted that in utero pigmentation [47] of cells and ciliation [104] of the RPE of the eye starts as early as E11.5 and E14.5, respectively. However, when recapitulating RPE development in vitro, pigmentation is generally observed in three to four weeks depending on the strategy employed. R. Sharma et al. explored the ciliation of RPEs by using PGE2 and found that ciliation is essential for RPE function in visual cycle. 

Eye field induction is the most important stage in RPE lineage trajectory as the RPE, the neural retina, the lens, and the optic stalk arise from the eye field that originates from the ANE. Few authors have focused on the derivation of eye field-like state from ANE for RPE development. However, critical transcription factors, such as RX [40,41] and Six3 [43,44], have been associated with normal eye development and in particular RPE development. Furthermore, during the course of optic cup development arising from the eye field, the outer layer forms RPE-receiving signals from the extraocular mesenchyme and the developing retina, as shown by the finding that problems in retina development lead to RPE formation as well [46]. Such an intricate process could not only be replicated in vitro with a few molecules targeting certain signalling pathways. It requires a more holistic and muti-factorial approach which the current developmental protocols do not cover extensively. This information can be used to enhance the expression of these transcription factors or use their expression efficiency quantitatively to validate the trajectory of RPE development. 

Further, epigenetic studies are quite important and should be considered in validating a protocol development apart from RNAseq-based characterization of the differentiation method. Inducing certain stages of RPE development using a cocktail of growth factors and small molecules leads to the activation of signalling pathways, but whether such activation also leads to changes in the epigenetic status of cells are not studied for all published protocols. Moreover, only modifying the epigenetic regulators can have significant effect on the cells. For example, in a study, *Xenopus* knockdown of a lysine-specific histone demethylase 5C (Kdm5c) resulted in small and deformed systems with abnormal RPEs [60]. 

The cellular aspect of RPE development is also crucial as it is known that RPE differentiation from the outer layer of the optic vesicle also depends on the factors secreted from the extraocular mesenchyme. It has been shown that in the presence of extraocular mesenchyme, RPE differentiation and maturation is promoted with a marked increase in Mitf expression [105]. However, in the absence of extraocular mesenchyme, the optic vesicle undergoes complete retinal transformation [106], thus underlining the role of the extraocular mesenchyme in RPE development. Although the effect of extraocular mesenchyme has been shown to be replicated by using the TGF family member Activin [105], it is still not completely known that if only Activin is essential for RPE development or if Activin along with other factors influences RPE differentiation. However, various protocols that have been published related to RPE differentiation do include Activin in the RPE specification stage. This could be possibly correlated with the extraocular mesenchyme; however, the specification of such specialized tissue not necessarily depends on just one factor as RPE and retinal development is highly coordinated and balanced. 

Thus, the development of a new methodology for improved and efficient differentiation of RPE should cover epigenetic factors, microRNAs, and secretome in comparable way to the native primary RPE. Such information can be derived from developmental studies as well as studies on the diseases and phenotypes of the RPE. This would also be beneficial in promoting cells for long-term functionality upon in vivo transplantation. 

## 6. Conclusions

RPE derivation using stem cells offers a plausible solution for the treatment of both dry and wet forms of AMD. The development of a defined and efficient protocol for RPE differentiation or the production of a highly pure population of iPSC-derived RPEs from the initial stages of development to the final maturation stage is still warranted. Using the knowledge derived from the basic cell biology of the RPE, and the information produced over the last three decades from developmental biology focusing on RPE and eye development using a variety of cell and animal models, has improved the design and development of strategies for iPSC-based RPE differentiation. Certain limitations still exist in producing functionally mature iPSC-derived RPEs and recapitulating the critical features, such as homogeneous pigmentation and ciliation, which still require further exploration. 

## Data Availability

Not applicable.
